# Supplementary material for: Analysis of PPARγ Signaling Activity in Psoriasis
Source: Int J Mol Sci. 2021 Aug 10;22(16):8603. doi: 10.3390/ijms22168603 (PMC8395241; doi:10.3390/ijms22168603)
Supplement: Supplementary file 1 [file ijms-22-08603-s001.zip › Supplemental materials_Analysis of PPARg signaling activity in psoriasis/Pathway models/Models images and html files/Model of PPARG related pathways in psoriasis (short version)/5639.html]

HIF1A


# Protein HIF1A

|  |  |
| --- | --- |
| URN | urn:agi-llid:3091 |
| Total Entities | 0 |
| Connectivity | 9987 |
| Name | HIF1A |
| Description | hypoxia inducible factor 1 alpha subunit |
| Notes | This gene encodes the alpha subunit of transcription factor hypoxia-inducible factor-1 (HIF-1), which is a heterodimer composed of an alpha and a beta subunit. HIF-1 functions as a master regulator of cellular and systemic homeostatic response to hypoxia by activating transcription of many genes, including those involved in energy metabolism, angiogenesis, apoptosis, and other genes whose protein products increase oxygen delivery or facilitate metabolic adaptation to hypoxia. HIF-1 thus plays an essential role in embryonic vascularization, tumor angiogenesis and pathophysiology of ischemic disease. Alternatively spliced transcript variants encoding different isoforms have been identified for this gene. [provided by RefSeq, Jul 2011] |
| Primary Cell Localization | Nucleus |
| Class | Transcription factor |
| ObjectType | Protein |

---

|  |  |
| --- | --- |
| Pathway | Proteins Involved in Stem Cell Exhaustion in Aging |
|  | Androgen Deficiency in Male Obesity |
|  | Hypoxia Inhibits mTOR Signaling |
|  | SIRT6 Signaling in Aging |
|  | P-cell Activation |
|  | Thyroid Hormones Common non-Genomic Effects in Hyperthyroidism |
|  | Androgens in Adipocyte Activation |
|  | T-Cell Maturation (Hypothesis) |
|  | Mast-Cell Activation via IgE Signaling |
|  | Macrophage Related Iron Uptake and Release |
|  | Negative Acute Phase Proteins Synthesis |
|  | Positive Acute Phase Proteins Synthesis |
|  | Adipokines Production by Adipocyte |
|  | IL1B Expression Targets -> Nociception |
|  | Circadian Clock in Sleep Regulation |
|  | Proteins Involved in Arterial Hypertension |
|  | Proteins Involved in Pulmonary Hypertension |
|  | Proteins Involved in Hypoxia-Induced Pulmonary Hypertension |
|  | Smooth Muscle Cell Dysfunction in Pulmonary Hypertension |
|  | Proteins Involved in Myocardial Ischemia |
|  | Proteins with Altered Expression in Melanoma |
|  | Melanoma |
|  | MITF as a Regulator of Melanoma Cell Development |
|  | Proteins Involved in Melanoma |
|  | Melanocyte Role in Skin Aging |
|  | Peripheral Tissue Microangiopathy |
|  | Endothelial Cell Dysfunction in Progressive Diabetic Nephropathy |
|  | Podocyte Dysfunction in Diabetic Nephropathy |
|  | Proteins Involved in Diabetic Nephropathy |
|  | Microangiopathy in Diabetic Neuropathy |
|  | Hepatic Stellate Cells in non-Alcoholic Fatty Liver Disease |
|  | Lipid Metabolism Impairement in non-Alcoholic Fatty Liver Disease |
|  | Oxidation of Fatty Acid in non-Alcoholic Fatty Liver Disease |
|  | Cardiovascular Effects in Hyperthyroidism |
|  | Adipokines Production by Adipocyte Impaired in Obesity |
|  | Proteins Involved in Obesity |
|  | Proteins Involved in Colorectal Neoplasms |
|  | Metastatic Colorectal Cancer |
|  | Peutz-Jeghers Syndrome |
|  | B-Cell Acute Lymphoblastic Leukemia |
|  | Osteoclast Activation in Multiple Myeloma |
|  | IL6/IGF1/VEGFA Signaling in Multiple Myeloma |
|  | Multiple Myeloma |
|  | T-Cell Acute Lymphoblastic Leukemia |
|  | Vascularization in Hepatocellular Carcinoma |
|  | Proteins Involved in Hepatocellular Carcinoma |
|  | WNT Signaling in Hepatocellular Carcinoma |
|  | Hepatocellular Carcinoma |
|  | Proteins Involved in Pancreatic Cancer |
|  | Goblet-Cell Related Mucus Secretion in Asthma |
|  | Mast-Cell Activation in Asthma |
|  | Mucin Production in Goblet Airway Epithelial Cells |
|  | Proteins Involved in Osteoarthritis |
|  | FXN Role in Friedreich Ataxia Progression |
|  | Proteins Involved in Epilepsy |
|  | Proteins Involved in Glioblastoma |
|  | Glioblastoma, Secondary |
|  | Astrocytoma |
|  | Proteins Involved in Glioma |
|  | Proteins Involved in Neuroblastoma |
|  | Proteins Involved in Amyotrophic Lateral Sclerosis |
|  | Proteins Involved in Vascular Dementia |
|  | Proteins Involved in Breast Cancer Related to ESR1 Signaling Pathway |
|  | Proteins Involved in Endometrial Cancer |
|  | Proteins Involved in Endometriosis |
|  | Clear Cell Ovarian Carcinoma |
|  | Ovarian Cancer |
|  | Proteins Involved in Clear Cell Ovarian Carcinoma |
|  | Proteins with Altered Expression in Ovarian Cancer |
|  | HPV Infection and Cancer |
|  | HPV E5 Regulates EDNRA and EGFR Signaling Pathway in Keratinocytes |
|  | Prostate Cancer |
|  | Androgen Receptor/c-Met Signaling in Prostate Cancer |
|  | FOXA2 Signaling in Prostate Cancer |
|  | Proteins Involved in Prostate Cancer |
|  | Netrin-1 Signaling in Age-Related Macular Degeneration |
|  | Oxidative Stress, All-Trans-Retinal and Lipofuscin Toxicity in AMD |
|  | HIF1A in Pathologic Ocular Neovascularization |
|  | VEGFA in Neovascular Glaucoma |
|  | Mucin Hyperproduction in Goblet and Mucous Cells |
|  | Endothelial Cell Dysfunction in Glomerulonephritis |
|  | Podocyte Dysfunction in Glomerulonephritis |
|  | Proteins with Altered Expression in Prostate Cancer |
|  | Endothelial Cell Dysfunction in Pyelonephritis |
|  | AREG -> HIF1A Expression Targets |
|  | Noradrenaline/Gs Expression Targets |
|  | Noreadrenaline/Gq Expression Targets |
|  | Elevated Receptors -> Expression Targets in Myocardium |
|  | CCL3 Expression Targets |
|  | CCL2 Expression Targets |
|  | EGF -> CTNN Expression Targets |
|  | EGF -> HIF1A Expression Targets |
|  | EGF -> AP-1/ATF Expression Targets |
|  | EGF -> CREB/CREBBP/ELK/SRF/MYC Expression Targets |
|  | EGF -> MEF/MYOD/NFATC Expression Targets |
|  | EGF -> STAT Expression Targets |
|  | EGFR/ERBB2 -> HIF1A Signaling |
|  | EREG -> HIF1A Expression Target |
|  | F2 -> AP-1/CREB/ELK/SRF/SP1 Expression Targets |
|  | F2 -> STAT1/NF-kB Expression Targets |
|  | FGF2 -> STAT Expression Targets |
|  | FGF2 -> AP-1/CREB/CREBBP/ELK/SRF/MYC Expression Targets |
|  | HBEGF -> HIF1A Expression Targets |
|  | HGF -> STAT Expression Targets |
|  | STAT3 and NFkB Activate Inflammation-Induced Tumorigenesis |
|  | Apoptosis Block by Inhibitor of Apoptosis Proteins (IAPs) in Cancer |
|  | Proteins with Altered Expression in Cancer-Associated Resisting to Cell Death |
|  | Suppression of Caspase Activity in Cancer |
|  | Apoptosis Evasion in Cancer: Overview |
|  | Hypoxia-Induced Cell Death Evasion in Cancer |
|  | Apoptosis Block by BCL2 Family Proteins in Cancer |
|  | HIF1A in Vasculogenic Mimicry of Cancer |
|  | VEGFA Dependent Angiogenesis in Cancer |
|  | FBLN5 Potentiates Migration and Proliferation of Endothelial Cells in Cancer |
|  | HIF1A as Master Regulator of Angiogenesis in Cancer |
|  | Proteins with Altered Expression in Cancer Metastases |
|  | Epithelial to Mesenchymal Transition in Cancer: Overview |
|  | BSG (CD147) in Cancer Cells Motility, Invasion and Survival |
|  | Metabolic Reprogramming in Cancer: Overview |
|  | Respiratory Chain Impairment in Cancer |
|  | Lactate as a Signaling Molecule in Cancer Cells |
|  | Metabolic Effects of Oncogenes and Tumor Suppressor in Cancer Cells |
|  | Mutations in Krebs Cycle Enzymes in Cancer |
|  | Glycolysis Activation in Cancer (Warburg Effect) |
|  | Proteins with Altered Expression in Cancer Metabolic Reprogramming |
|  | Treg Cells Promote Immunosuppression in Cancer Immune Escape |
|  | Adenosine/cAMP Promote Immunosuppression by Treg Cells |
|  | EZH2 Methyltransferase Role in Cancer |
|  | IGF1 -> MEF/MYOD/MYOG Expression Targets |
|  | IGF2 -> STAT Expression Targets |
|  | IGF1 -> STAT Expression Targets |
|  | IGF2 -> HIF1A/MYC Expression Targets |
|  | IGF1 -> ELK/SRF/HIF1A/MYC/SREBF Expression Targets |
|  | Insulin -> STAT Expression Targets |
|  | Insulin -> CEBPA/CTNNB/FOXA/FOXO Expression Targets |
|  | Insulin -> MEF/MYOD Expression Targets |
|  | Insulin -> ELK/SRF/HIF1A/MYC/SREBF Expression Targets |
|  | IGF1R -> ELK/SRF/HIF1A/MYC/SREBF Signaling |
|  | EGFR -> Expression Targets in Skin |
|  | NOTCH -> Expression Targets in Skin |
|  | IFNG/IFNR Expression Targets |
|  | IL3 Expression Targets |
|  | IL6 Expression Targets |
|  | Prostaglandin F Expression Targets |
|  | LPA Expression Targets |
|  | S1P Expression Targets |
|  | TNF Receptors -> Expression Targets in Lymphoid System and Blood |
|  | NRG1 -> EP300/ETS/ETV/SP1 Expression Targets |
|  | NRG1 -> CREB/CREBBP/ELK/SRF/MYC Expression Targets |
|  | NRG1/Catenin Expression Targets |
|  | NRG1 -> STAT Expression Targets |
|  | NRG1 -> AP-1/ATF Expression Targets |
|  | NRG1 -> HIF1A Expression Target |
|  | Elevated Receptors -> Expression Targets in Nerve Tissue |
|  | BDNF Expression Targets |
|  | Adenosine Expression Targets |
|  | PDGF -> AP-1/CREB/CREBBP/MYC Expression Targets |
|  | PDGF -> STAT Expression Targets |
|  | AGT -> CREB Expression Targets |
|  | TAC1 Expression Targets |
|  | AGT -> STAT Expression Targets |
|  | PRL/PRLR Expression Targets |
|  | PRL/GHR -> STAT Expression Targets |
|  | PRL/GHR -> NF/kB/ELK/SRF/MYC Expression Targets |
|  | CSF1 -> STAT Expression Targets |
|  | KITLG -> STAT Expression Targets |
|  | KITLG -> AP-1/CREB/CREBBP/MYC Expression Targets |
|  | CSF1 -> AP-1/CREB/CREBBP/MYC Expression Targets |
|  | PGF -> AP-1/CREB/CREBBP/MYC Expression Targets |
|  | Elevated Receptors -> Expression Targets in Breast |
|  | HIF1 Signaling |
|  | TGFA -> HIF1A Expression Targets |
|  | TGFB1-TGFBR2 Expression Targets |
|  | TGFB1-TGFBR1/AP-1 Expression Targets |
|  | TGFB1-ACVRL1 Expression Targets |
|  | TNF -> NF-kB Expression Targets |
|  | TNF -> AP-1 Expression Targets |
|  | TNF -> STAT Expression Targets |
|  | Thrombopoietin -> AP-1/CREB/CREBBP/MYC Expression Targets |
|  | Thrombopoietin -> STAT Expression Targets |
|  | IL1B -> NO Expression Targets |
|  | TLR4 -> AP-1/EGR1/HIF1A Expression Targets |
|  | IL1B -> PGE2 Expression Targets |
|  | TLR1 -> 2/6 Expression Targets |
|  | IL1B Expression Targets |
|  | IL1A Expression Targets |
|  | Mitochondrial Damage Induced by Hypoxia |
|  | Elevated Receptors -> Expression Targets in Kidney |
|  | pprarg neg, uknown targets, ps-positive |
|  | Model of PPARG signaling in psoriasis |
|  | PPARG negative regulators and targets |
|  | Model of PPARG related pathways in psoriasis (short version) |
|  | New Pathway |
|  | 1\_TNF and IL1B provoke ECM degradation in osteoarthritis |
|  | 2\_Chondrocyte death in osteoarthritis |
|  | 2\_The role of endothelial cells in pyelonephritis |
|  | 2\_Podocyte dysfunction in glomerulonephritis |
|  | 2\_Oxidative stress, all-trans-retinal, and lipofuscin toxicity in agerelated macular degeneration |
|  | 3\_1\_Mucus accumulation in asthma |
|  | 4\_Mucin hyperproduction in goblet and mucous cells in COPD |
|  | 3\_Human papilloma virus E4 and E5 proteins in viral reproduction |
|  | 1\_Microangiopathy in diabetic neuropathy |
|  | drug-target pairs direct regulation |
|  | drug-target molsynthesis |

---

|  |  |
| --- | --- |
| Group | Proteins Involved in Cowden Syndrome |

---

|  |  |
| --- | --- |
| MedScan ID | 3091 |

---

|  |  |
| --- | --- |
| LocusLink ID | 3091 |
|  | 15251 |
|  | 29560 |

---

|  |  |
| --- | --- |
| Alias | bHLHe78 |
|  | MOP I |
|  | hypoxia inducible factor 1, alpha subunit |
|  | PAS domain-containing protein 8 |
|  | basic-helix-loop-helix-PAS protein MOP1 |
|  | LOC115049 |
|  | member of PAS superfamily I |
|  | HIF1alpha |
|  | ARNT interacting protein |
|  | AA959795 |
|  | Hypoxia-inducible factor 1 alpha |
|  | hypoxia-inducible factor 1 alpha isoform I.3 |
|  | class E basic helix-loop-helix protein 78 |
|  | PASD8 |
|  | OTTHUMP00000179063 |
|  | hypoxia-inducible factor 1, alpha subunit (basic helix-loop-helix transcription factor) |
|  | Hypoxia-inducible factor 1a |
|  | Member of PAS protein I |
|  | member of PAS protein 1 |
|  | member of PAS superfamily 1 |
|  | OTTMUSP00000021323 |
|  | hypoxia inducible factor 1 subunit alpha |
|  | ARNT-interactive protein |
|  | ARNT interactive protein |
|  | HIF1A |
|  | hypoxia-inducible factor 1 DNA-binding subunit |
|  | member of PAS protein-1 |
|  | ARNT-interacting protein |
|  | HIF1 |
|  | MOP1 |
|  | HIF-1A |
|  | HIF-1alpha |
|  | HIF1-ALPHA |
|  | HIF-1-alpha |
|  | hypoxia inducible factor 1 alpha subunit |
|  | hypoxia inducible factor 1, alpha subunit (basic helix-loop-helix transcription factor) |
|  | hypoxia-inducible factor1alpha |
|  | hypoxia-inducible factor 1-alpha |
|  | HIF-1 alpha |
|  | HIF-1 |
|  | HIF I |
|  | hypoxia-inducible factor 1 |
|  | HIF1 alpha |
|  | hypoxia-inducible factor I |

---

|  |  |
| --- | --- |
| GO ID | 0001228 |
|  | 0003700 |
|  | 0000981 |
|  | 0070888 |
|  | 0051879 |
|  | 0000977 |
|  | 0019899 |
|  | 0035035 |
|  | 0042826 |
|  | 0035257 |
|  | 0002039 |
|  | 0019904 |
|  | 0046982 |
|  | 0019901 |
|  | 0043565 |
|  | 0008134 |
|  | 0031625 |
|  | 0001922 |
|  | 0001525 |
|  | 0019896 |
|  | 0003208 |
|  | 0051216 |
|  | 0006879 |
|  | 0071456 |
|  | 0071347 |
|  | 0021987 |
|  | 0032963 |
|  | 0002248 |
|  | 0019221 |
|  | 0048546 |
|  | 0071542 |
|  | 0051541 |
|  | 0035162 |
|  | 0001892 |
|  | 0061030 |
|  | 0001837 |
|  | 0042593 |
|  | 0001947 |
|  | 0042541 |
|  | 0097411 |
|  | 0060574 |
|  | 0061072 |
|  | 0006089 |
|  | 0007595 |
|  | 0042789 |
|  | 0046716 |
|  | 0032007 |
|  | 0030502 |
|  | 0010629 |
|  | 0045926 |
|  | 2001054 |
|  | 1903377 |
|  | 2000378 |
|  | 0070244 |
|  | 0001755 |
|  | 0021502 |
|  | 0003151 |
|  | 0032364 |
|  | 0045766 |
|  | 1903599 |
|  | 0043536 |
|  | 0032722 |
|  | 0070101 |
|  | 0001938 |
|  | 0010634 |
|  | 0045648 |
|  | 0010628 |
|  | 0045821 |
|  | 0046886 |
|  | 0035774 |
|  | 0016239 |
|  | 0002052 |
|  | 0051000 |
|  | 1902895 |
|  | 0010870 |
|  | 0045944 |
|  | 0061419 |
|  | 0045893 |
|  | 0010575 |
|  | 0030949 |
|  | 0043687 |
|  | 0016579 |
|  | 0016567 |
|  | 1903715 |
|  | 0010468 |
|  | 0006357 |
|  | 0061418 |
|  | 0043619 |
|  | 0006355 |
|  | 0032909 |
|  | 0001666 |
|  | 0010039 |
|  | 0014850 |
|  | 0061298 |
|  | 0007165 |
|  | 0006366 |
|  | 0010573 |
|  | 0008542 |
|  | 0090575 |
|  | 1904115 |
|  | 0005737 |
|  | 0005829 |
|  | 0031514 |
|  | 0016604 |
|  | 0000790 |
|  | 0016607 |
|  | 0005654 |
|  | 0005634 |
|  | 0032991 |
|  | 0005667 |
|  | 0003677 |
|  | 0044877 |
|  | 0001568 |
|  | 0048514 |
|  | 0048593 |
|  | 0030154 |
|  | 0043066 |
|  | 0043524 |
|  | 0030279 |
|  | 0000122 |
|  | 0045906 |
|  | 0043065 |
|  | 0010508 |
|  | 0008284 |
|  | 0045722 |
|  | 0048661 |
|  | 0050790 |
|  | 0042127 |
|  | 0006110 |
|  | 0070243 |
|  | 0060992 |
|  | 0001944 |
|  | 0006953 |
|  | 0071245 |
|  | 0071279 |
|  | 1903928 |
|  | 0035690 |
|  | 0071257 |
|  | 0071333 |
|  | 0070301 |
|  | 0032869 |
|  | 0071482 |
|  | 0071396 |
|  | 0071222 |
|  | 0071260 |
|  | 0071250 |
|  | 0071407 |
|  | 0097237 |
|  | 0060135 |
|  | 0045793 |
|  | 1900037 |
|  | 0010165 |
|  | 0014823 |
|  | 0043279 |
|  | 0072347 |
|  | 0010996 |
|  | 0032025 |
|  | 0042493 |
|  | 0032355 |
|  | 0043627 |
|  | 0051384 |
|  | 0009749 |
|  | 0009612 |
|  | 0014074 |
|  | 0009651 |
|  | 0030424 |
|  | 0005623 |
|  | 0005515 |
|  | 0000989 |
|  | 0000988 |
|  | 0003690 |
|  | 0009434 |
|  | 0003705 |
|  | 0044212 |
|  | 0004871 |
|  | 0005730 |
|  | 0032403 |
|  | 0006351 |
|  | 0001077 |
|  | 0001076 |
|  | 0007219 |
|  | 0042981 |
|  | 0034244 |
|  | 0033554 |
|  | 2001141 |

---

|  |  |
| --- | --- |
| KEGG ID | hsa:3091 |
|  | mmu:15251 |
|  | rno:29560 |

---

|  |  |
| --- | --- |
| Organism | Homo sapiens {Organism urn:agi-taxid:9606} |
|  | Mus musculus {Organism urn:agi-taxid:10090} |
|  | Rattus norvegicus {Organism urn:agi-taxid:10116} |
|  | Homo sapiens |
|  | Mus musculus |
|  | Rattus norvegicus |

---

|  |  |
| --- | --- |
| Mouse chromosome position | 12 31.99 cM |
|  | 12 31.0 cM |

---

|  |  |
| --- | --- |
| OMIM ID | 603348 |

---

|  |  |
| --- | --- |
| Rat chromosome position | 6q24 |

---

|  |  |
| --- | --- |
| Hugo ID | 4910 |
|  | HGNC:4910 |

---

|  |  |
| --- | --- |
| Human chromosome position | 14q23.2 |

---

|  |  |
| --- | --- |
| Swiss-Prot Accession | Q16665 |
|  | D0VY79 |
|  | Q16665.1 |
|  | Q61221 |
|  | A0A0R4J1E9 |
|  | Q3UCW2 |
|  | Q61221.3 |
|  | O35800 |
|  | D4A8P8 |
|  | O35800.1 |
|  | C0LZJ3 |
|  | Q53XP6 |
|  | Q96PT9 |
|  | Q9UPB1 |
|  | O08741 |
|  | O08993 |
|  | Q61664 |
|  | Q61665 |
|  | Q8C681 |
|  | Q8CC19 |
|  | Q8CCB6 |
|  | Q8R385 |
|  | Q9CYA8 |
|  | Q9WTU9 |
|  | A8MYV6 |

---

|  |  |
| --- | --- |
| PIR ID | I38972 |
|  | JC4837 |
|  | JC5809 |

---

|  |  |
| --- | --- |
| GenBank ID | NC\_000014 |
|  | NM\_001530 |
|  | NP\_001521 |
|  | NM\_181054 |
|  | NP\_851397 |
|  | NM\_001243084 |
|  | NP\_001230013 |
|  | NG\_029606 |
|  | AF208487 |
|  | AAF20149 |
|  | AH006957 |
|  | AAC68568 |
|  | AL137129 |
|  | CH471061 |
|  | EAW80806 |
|  | EAW80807 |
|  | EAW80808 |
|  | EAW80809 |
|  | AB073325 |
|  | BAB70608 |
|  | AB500182 |
|  | BAI49183 |
|  | AB733094 |
|  | BAM28632 |
|  | AF207601 |
|  | AAF20139 |
|  | AF207602 |
|  | AAF20140 |
|  | AF304431 |
|  | AAG43026 |
|  | AJ227916 |
|  | AK093749 |
|  | AK296876 |
|  | BAG59438 |
|  | AK299551 |
|  | BAG61496 |
|  | AK304315 |
|  | BAG65167 |
|  | AK304436 |
|  | BAG65259 |
|  | AK312397 |
|  | BAG35314 |
|  | AU123241 |
|  | BC012527 |
|  | AAH12527 |
|  | BT009776 |
|  | AAP88778 |
|  | BX648795 |
|  | CN264295 |
|  | CN264320 |
|  | DC344032 |
|  | DQ975378 |
|  | FJ790247 |
|  | ACN88547 |
|  | U22431 |
|  | AAC50152 |
|  | U29165 |
|  | AAC51210 |
|  | X72726 |
|  | Q16665 |
|  | NC\_000078 |
|  | NM\_001313920 |
|  | NP\_001300849 |
|  | NM\_001313919 |
|  | NP\_001300848 |
|  | NM\_010431 |
|  | NP\_034561 |
|  | XM\_006515478 |
|  | XP\_006515541 |
|  | XM\_017314961 |
|  | XP\_017170450 |
|  | AC124712 |
|  | AH006789 |
|  | AAC53461 |
|  | CH466526 |
|  | EDL36500 |
|  | EDL36501 |
|  | U59496 |
|  | AAC52730 |
|  | Y09085 |
|  | CAA70305 |
|  | CAA70306 |
|  | Y09086 |
|  | Y13656 |
|  | AF003695 |
|  | AAC53455 |
|  | AK008499 |
|  | AK017853 |
|  | BAB30975 |
|  | AK033471 |
|  | BAC28305 |
|  | AK034087 |
|  | BAC28578 |
|  | AK048798 |
|  | AK076395 |
|  | BAC36320 |
|  | AK138944 |
|  | BAE23829 |
|  | AK150367 |
|  | BAE29500 |
|  | BC026139 |
|  | AAH26139 |
|  | BY725437 |
|  | CO810618 |
|  | CX241289 |
|  | X95002 |
|  | CAA64458 |
|  | X95580 |
|  | CAA64833 |
|  | Q61221 |
|  | NC\_005105 |
|  | XM\_006240196 |
|  | XP\_006240258 |
|  | XM\_006240197 |
|  | XP\_006240259 |
|  | XM\_006240198 |
|  | XP\_006240260 |
|  | NM\_024359 |
|  | NP\_077335 |
|  | XM\_006240199 |
|  | XP\_006240261 |
|  | AC\_000074 |
|  | AAHX01045099 |
|  | AC134165 |
|  | CH473947 |
|  | EDM03621 |
|  | EDM03622 |
|  | AF057308 |
|  | AAD24413 |
|  | FQ215710 |
|  | Y09507 |
|  | CAA70701 |
|  | O35800 |
|  | XM\_005267573 |
|  | XM\_005267574 |
|  | ABBA01018302 |
|  | XP\_006515540 |
|  | XP\_005267630 |
|  | XP\_005267631 |
|  | AAHY01101338 |
|  | AC\_000146 |
|  | XP\_011242298 |
|  | XP\_011242299 |
|  | AC\_000034 |
|  | AF004155 |
|  | AF004153 |
|  | AF004154 |
|  | XM\_008764695 |
|  | NC\_018925 |
|  | XP\_008762917 |
|  | XM\_011243996 |
|  | XM\_011243997 |
|  | XM\_006515477 |
|  | AABR06044850 |
|  | AF050115 |
|  | AF050118 |
|  | AF050119 |
|  | AF004141 |
|  | AF050116 |
|  | AF050117 |
|  | AMYH02029316 |
|  | AF050121 |
|  | AF004148 |
|  | AF050122 |
|  | AF004149 |
|  | AF004146 |
|  | AF050120 |
|  | AF004147 |
|  | AF050125 |
|  | AF004144 |
|  | AF050126 |
|  | AF004145 |
|  | AF050123 |
|  | AF004142 |
|  | AF050124 |
|  | AF004143 |
|  | AF004151 |
|  | AF004152 |
|  | AF050127 |
|  | AF004150 |

---

|  |  |
| --- | --- |
| Swiss-Prot ID | HIF1A\_HUMAN |
|  | HIF1A\_MOUSE |
|  | HIF1A\_RAT |

---

|  |  |
| --- | --- |
| Cell Localization | Cytoplasm |
|  | Nucleus |
|  | Nucleus speckle |

---

|  |  |
| --- | --- |
| Ensembl ID | ENSG00000100644 |
|  | ENSP00000437955.1 |
|  | ENST00000539097.2 |
|  | ENSP00000338018.4 |
|  | ENST00000337138.9 |
|  | ENSP00000323326.6 |
|  | ENST00000323441.10 |
|  | ENSMUSG00000021109 |
|  | ENSMUSP00000106088.1 |
|  | ENSMUST00000110461.7 |
|  | ENSMUSP00000021530.7 |
|  | ENSMUST00000021530.7 |
|  | ENSRNOG00000008292 |
|  | ENSRNOP00000070177.1 |
|  | ENSRNOT00000077935.1 |
|  | ENSRNOP00000042230.3 |
|  | ENSRNOT00000049725.3 |
|  | ENSRNOP00000070177 |
|  | ENST00000539097 |
|  | ENST00000337138 |
|  | ENSMUST00000110461 |
|  | ENST00000337138.8 |
|  | ENSP00000437955 |
|  | ENST00000323441 |
|  | ENSP00000323326 |
|  | ENSMUSP00000021530 |
|  | ENSMUSP00000106088 |
|  | ENSRNOP00000042230 |
|  | ENSMUST00000021530 |
|  | ENSRNOT00000077935 |
|  | ENSP00000338018 |
|  | ENSP00000378446 |
|  | ENST00000394997 |
|  | ENSRNOT00000049725 |

---

|  |  |
| --- | --- |
| MGI ID | MGI:106918 |
|  | 106918 |

---

|  |  |
| --- | --- |
| RGD ID | 61928 |

---

|  |  |
| --- | --- |
| Unigene ID | Rn.10852 |
|  | Hs.597216 |
|  | Mm.3879 |
|  | Hs.719495 |
|  | Mm.397466 |
|  | Mm.446610 |

---

|  |  |
| --- | --- |
| Homologene ID | 1171 |

---

|  |  |
| --- | --- |
| Shape | O-vertex |

---

|  |  |
| --- | --- |
| IPI ID | IPI00406683 |
|  | IPI00336700 |
|  | IPI00951313 |
|  | IPI00003442 |
|  | IPI00332963 |

---
